# Effect of the skincare product on facial skin microbial structure and biophysical parameters: A pilot study

**DOI:** 10.1002/mbo3.1236

**Published:** 2021-10-06

**Authors:** Bo Kyoung Hwang, Sado Lee, Joonoh Myoung, Seung Jin Hwang, Jun Man Lim, Eui Taek Jeong, Sun Gyoo Park, Sung Hun Youn

**Affiliations:** ^1^ R&D Center LG Household and Health Care Ltd Seoul South Korea

**Keywords:** skin environment, skin microbiome, skin physiology, skincare product

## Abstract

Daily use of cosmetics is known to affect the skin microbiome. This study aimed to determine the bacterial community structure and skin biophysical parameters following the daily application of a skincare product on the face. Twenty‐five Korean women, who used the same skincare product for four weeks participated in the study. During this period, skin hydration, texture, sebum content, and pH were measured, and skin swab samples were collected on the cheeks. The microbiota was analyzed using the MiSeq system. Through these experiments, bacterial diversity in facial skin increased and the microbial community changed after four weeks of skincare product application. The relative abundance of *Cutibacterium* and *Staphylococcus* increased, significant changes in specific bacterial modules of the skin microbial network were observed, and skin hydration and texture improved. It was suggested that daily use of skincare products could affect the microbial structure of facial skin as well as the biophysical properties of the facial skin. These findings expand our understanding of the role of skincare products on the skin environment.

## Graphical abstract

The daily use of skincare products affects both the facial skin microbial structure and its biophysical properties. Based on the canonical correspondence analysis plot, after the use of a skincare product, the skin microbiomes were significantly separated from the initial skin microbiome. Moreover, the study showed a significant correlation between skin microbial community and skin biophysical parameters such as skin hydration and texture, suggesting a relationship between these factors.

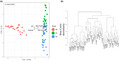

## 1 INTRODUCTION

The skin is an ecosystem comprising various host structures and colonizing microorganisms, including bacteria, fungi, and viruses. Its composition is unique to each person and part of the body (Byrd et al., 2018; Perez et al., 2016). Through evolution, skin microorganisms have adapted to individual host environments and cells. On the one hand, the skin provides nutrients and abiotic factors (e.g., temperature and humidity) that let skin microorganisms grow (Findley et al., 2013; Kong, 2011); on the other hand, these microorganisms prevent the colonization of pathogens, directly and indirectly benefiting the host (Schommer & Gallo, 2013).

The development of next‐generation sequencing techniques has facilitated the study of the human microbiome, first in the gut and later on also on the skin. As a result, inter‐personal or intra‐personal skin microbiome diversity has been revealed. Specifically, the structure of the skin microbiome has been seen to vary depending on the environment (Kim et al., 2018; Leung et al., 2020), gender (Ross et al., 2017; Ying et al., 2015), race (Li et al., 2019), and age (Kim et al., 2019; Shibagaki et al., 2017), as well as over time. Several studies have shown that long‐term stability reflects the initial status of the microbiome and the host's specific lifestyle (Flores et al., 2014; Grice et al., 2009; Hillebrand et al., 2021; Oh et al., 2016). Furthermore, a balanced skin microbiome is known to play an important role in skin health, as any alterations lead to the overgrowth of pathogenic strains linked to various skin diseases (Kim et al., 2018; Kong et al., 2012; Williams & Gallo, 2015). The daily use of cosmetics might also affect the skin microbiome, and may be determined by product type, duration of use, and participant characteristics (Ciardiello et al., 2020; Lee et al., 2018; Two et al., 2016; Wallen‐Russell, 2019). While most research in this sense has focused on microbial diversity or changes to individual bacterial strains, little is known about the impact of cosmetics on the overall microbial structure.

In the present study, the whole microbial community structure was analyzed, and biophysical parameters of the skin were measured following the use of a skincare product in Korean women. The co‐occurrence network between bacterial community and skin biophysical parameters offers a broad understanding of the role of cosmetics on the skin ecosystem.

## 2 MATERIALS AND METHODS

### 2.1 Participant recruitment and study design

**FIGURE A1:**
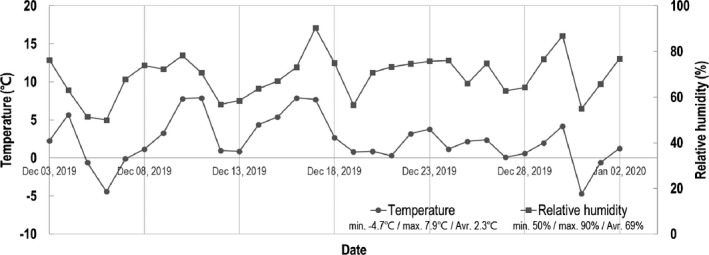
Temperature and relative humidity in Daejeon, South Korea during this study. Data were obtained from KMA (Korea Meteorological Administration) National Climate Data Center (https://data.kma.go.kr/cmmn/main.do).

Twenty‐five healthy Korean women between 30 and 58 years of age, and residing in Daejeon were recruited in this study (average age: 43 years). Participants who met the following conditions were excluded: (1) were pregnant or lactating; (2) had a lesion like spots, acne, erythema, or atopic dermatitis at the test site; (3) had infectious skin disease; (4) were sensitive to cosmetics, pharmaceuticals, or daily exposure to light; and (5) had undergone skin treatment (scaling, fillers, botox, laser treatment, etc.) within 3 months. The essence type of a moisturizing skincare product (su:m37° Secret Essence, LG Household & Health Care Ltd) was provided to all volunteers and the ingredients were listed in Table A1. Participants were asked to apply the skincare product on their face twice a day (morning and evening) after facial washing with their cleanser for four weeks. They were allowed to maintain their own skincare routines except for prohibiting the use of antibiotics, steroids, and cosmetics with similar formulations or ingredients to the target product. Swab sampling and measurements of skin biophysical parameters were performed on the cheek (previously unwashed for at least 8 h) three times during the experiment: before the use of skincare product (T0), and two (T2) and four (T4) weeks after. Before measurements, the participants relaxed under constant temperature and humidity conditions (indoor temperature 20–25°C, humidity 40–60%) for at least 30 min while the weather was dry and cold during this study (Figure A1).

### 2.2 Measurements of skin biophysical parameters

Skin biophysical parameters, including hydration, texture, sebum content, and pH, were assessed. Skin hydration levels were measured using a Corneometer^®^ (Courage+Khazaka electronic GmbH, Köln, Germany) and expressed as arbitrary units (A.U.). Facial skin texture was analyzed with a Visioscan® camera (Courage+Khazaka electronic GmbH) and expressed as SEr (roughness) values. Sebum content was measured using the Visioscan® camera and Sebufix® F 16 foil (Courage+Khazaka electronic GmbH) and was expressed as Area %. Lastly, facial pH was measured with a skin pH meter (Courage+Khazaka electronic GmbH).

### 2.3 DNA extraction from human skin samples

Skin swabbing of the cheek was performed with sterile swabs (ESwab^®^; COPAN Diagnostics Inc.) following the procedure described by Dimitriu et al. (Dimitriu et al., 2019). The supernatant was centrifuged at 15,814 *g* for 5 min at 4°C. Bacterial DNA was extracted from the collected samples using a DNA extraction kit (PowerSoil DNA Isolation Kit; MO BIO, QIAGEN) following the manufacturer's instructions.

### 2.4 Amplification of the V3‐4 region of the 16S rRNA gene

To analyze the microbiome community, the variable V3‐4 region (approximately 400–500 bp) of the 16S ribosomal RNA (rRNA) gene was amplified. The primer sets used for PCR amplification of target genes in the V3‐4 region are listed in Table 1. The PCR reaction mixture (25 μl total volume) contained 10 ng of DNA template, 2.5 μl of 16S‐v34 Fs (5.0 μM) primers, 2.5 μl of 16S‐v34‐R (5.0 μM) primers, and 12.5 μl of 2X KAPA HiFi HotStart ReadyMix (Roche, Midrand, South Africa). The PCR thermal profile consisted of an initial denaturation step at 96°C for 3 min, followed by 25 cycles at 96°C for 30 s, 55°C for 30 s, and 72°C for 30 s, as well as a final step at 72°C for 7 min. The PCR products were cleaned with AMPure XP beads (Beckman Coulter) using a 1.4× ratio, quantified using Picogreen fluorescence (Life Technologies), and volume‐adjusted prior to the second round of PCR. The PCR products were used to construct 16S rDNA gene libraries according to guidelines for sequencing on the MiSeq System (Illumina Inc.).

**TABLE 1:** Primer sequences for the 1^st^ PCR

V3‐4 region	Sequence (5′→3′)
16S v34_F	TCGTCGGCAGCGTCAGATGTGTATAAGAGACAGCCTACGGGNGGCWGCAG
16S v34_F_N1	TCGTCGGCAGCGTCAGATGTGTATAAGAGACAGNCCTACGGGNGGCWGCAG
16S v34_F_N2	TCGTCGGCAGCGTCAGATGTGTATAAGAGACAGNNCCTACGGGNGGCWGCAG
16S v34_F_N3	TCGTCGGCAGCGTCAGATGTGTATAAGAGACAGNNNCCTACGGGNGGCWGCAG
16S v34_R	GTCTCGTGGGCTCGGAGATGTGTATAAGAGACAGGACTACHVGGGTATCTAATCC
16S v34_R_N1	GTCTCGTGGGCTCGGAGATGTGTATAAGAGACAGNGACTACHVGGGTATCTAATCC
16S v34_R_N2	GTCTCGTGGGCTCGGAGATGTGTATAAGAGACAGNNGACTACHVGGGTATCTAATCC
16S v34_R_N3	GTCTCGTGGGCTCGGAGATGTGTATAAGAGACAGNNNGACTACHVGGGTATCTAATCC

### 2.5 Analysis of sequencing data

The obtained sequences were analyzed using QIIME 1.9.1 (Caporaso et al., 2012; Kuczynski et al., 2012; Navas‐Molina et al., 2013). Paired sequences were merged, and low‐quality sequences and chimeric reads were removed using USEARCH (Edgar, 2010, 2017) and the VSEARCH pipeline (Rognes et al., 2016). Next, the sequences were clustered into operational taxonomic units (OTUs) based on 97% sequence identity with the Silva 132 (Quast et al., 2013) and NCBI (Chen et al., 2010) databases using PyNAST (Caporaso et al., 2010). Each OTU was assigned a taxonomy ID based on the Silva and NCBI databases using RDP classifiers (Soergel et al., 2012; Wang et al., 2007). The alpha_diversity.py program of QIIME was applied to analyze alpha diversity (Gotelli & Colwell, 2001). Differences between samples were evaluated using the Mann‐Whitney *U*‐test and Kruskal–Wallis test in R. Statistical significance was set at *p* < 0.05.

Canonical correspondence analysis (CCA) was performed to assess the correlation between skin biophysical parameters and microbial composition using the Bray‐Curtis distance matrix in R. Significance was evaluated using the permutation test in R. Spearman's correlation coefficient was applied to evaluate the correlation among bacteria or between the skin biophysical parameters and bacteria. OTUs with less than 25% prevalence in each group were trimmed. Microbial networks were constructed using the Gephi program (Bastian et al., 2009), with the following criteria: threshold = 0.6 and adjusted *p* < 0.05. The modularity of the bacterial network was visualized by a heatmap using the pheatmap package in R. The correlation between bacteria and skin biophysical parameters (Spearman's correlation threshold = 0.4, adjusted *p* < 0.05) was visualized in Cytoscape (Shannon et al., 2003). Bacterial profiles in each sampling group were compared by linear discriminant analysis effect size (LEfSe).

## 3 RESULTS

### 3.1 Microbial communities in facial skin

**FIGURE 1:**
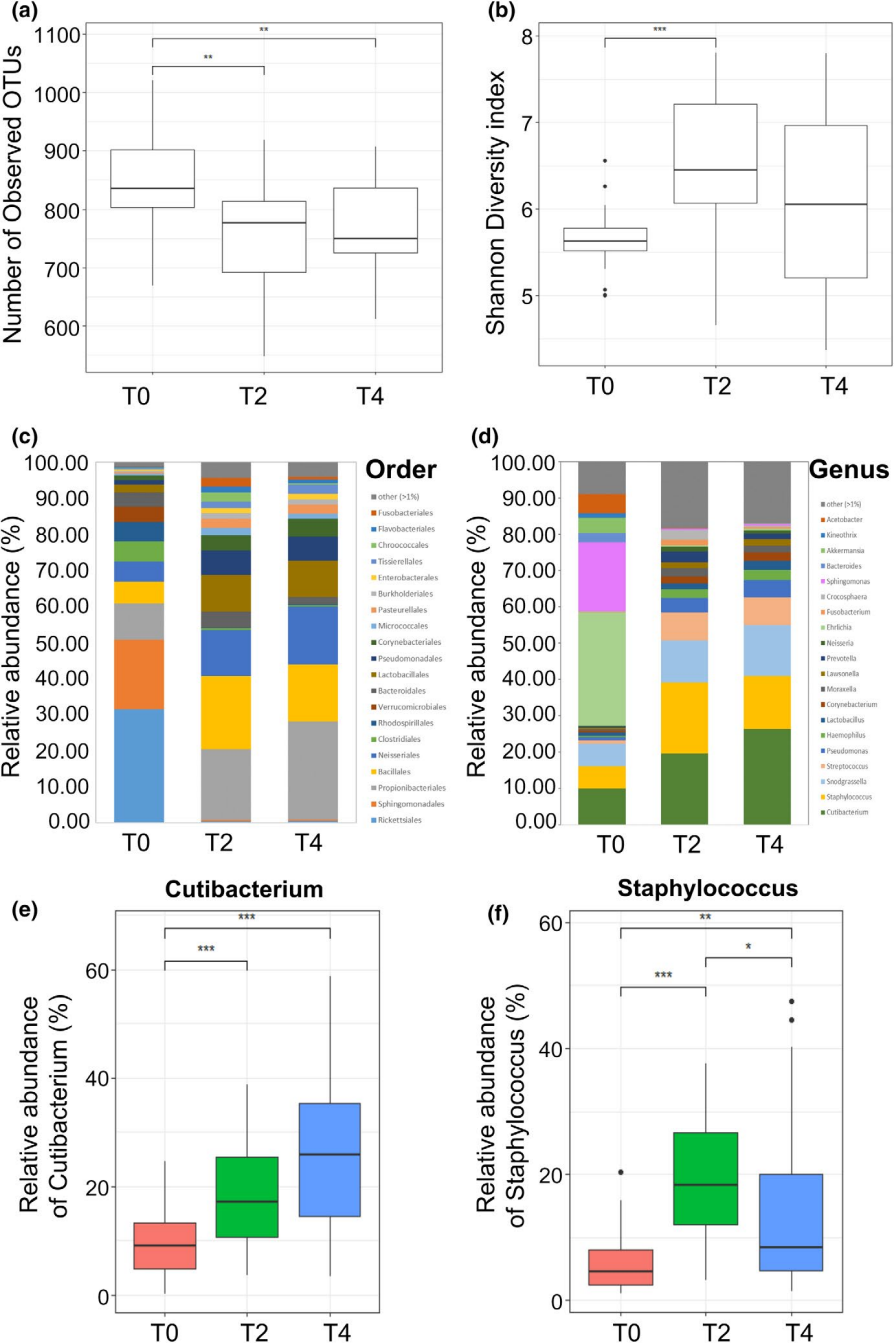
Comparison of skin microbiome composition following use of the skincare product. (a) The number of observed OTUs. (b) Shannon diversity index. Relative abundance of bacteria at (c) order level and (d) genus level. Relative abundance of (e) *Cutibacterium* and (f) *Staphylococcus* in skin sample. *p < 0.05, **p < 0.01, ***p < 0.005

A total of 8,872,604 reads for 75 samples were analyzed after trimming (Table A2). The number of observed OTUs was higher in T0 samples (average 840 ± 93.08) than in T2 (759 ± 83.58) and T4 (765.64 ± 75.80) samples (Figure 1a). The average Shannon diversity index was higher in T2 samples (6.53 ± 0.82) than in T0 and T4 samples (5.64 ± 0.35 and 6.04 ± 0.98, respectively) (Figure 1b). The most abundant phyla in skin samples were Proteobacteria and Firmicutes, followed by Actinobacteria.

Microbiota composition was compared at the order and genus level (Figure 1c,d). The dominant orders differed with sampling time (Figure 1c), particularly between T0 samples and those collected after use of the skincare product (T2 and T4). Before treatment, Rickettsiales was the main order (average 31.57%) but was rarely found after treatment (< 1% in T2 and T4). In contrast, Bacillales, a common order in human skin, was more abundant in T2 samples (20.30%) than T0 (6.92%) and T4 (15.79%) samples. The commensal bacteria Propionibacteriales were similarly increased after treatment (T0, 10.07%; T2, 19.56%; T4, 27.14%). At the genus level, microbiota composition changed following the use of the skincare product (Figure 1d). *Ehrlichia*, *Sphingomonas*, and *Cutibacterium* were the dominant genera in the T0 sample. In contrast, *Cutibacterium*, *Staphylococcus*, *Snodgrassella*, and *Streptococcus* became the dominant genera in T2 and T4 samples. Specifically, the relative abundance of *Cutibacterium* and *Staphylococcus*, both of which are commonly found in human skin, increased from 10.01% (T0) to 19.61% (T2) and 26.41% (T4) (Figure 1e, *p* < 0.005) and from 6.08% (T0) to 19.59% (T2) and 14.62% (T4) (Figure 1f, *p* < 0.005, *p* < 0.01, *p* < 0.05), respectively.

### 3.2 Dynamic shifts in microbial structures following the use of the skincare product

**FIGURE 2:**
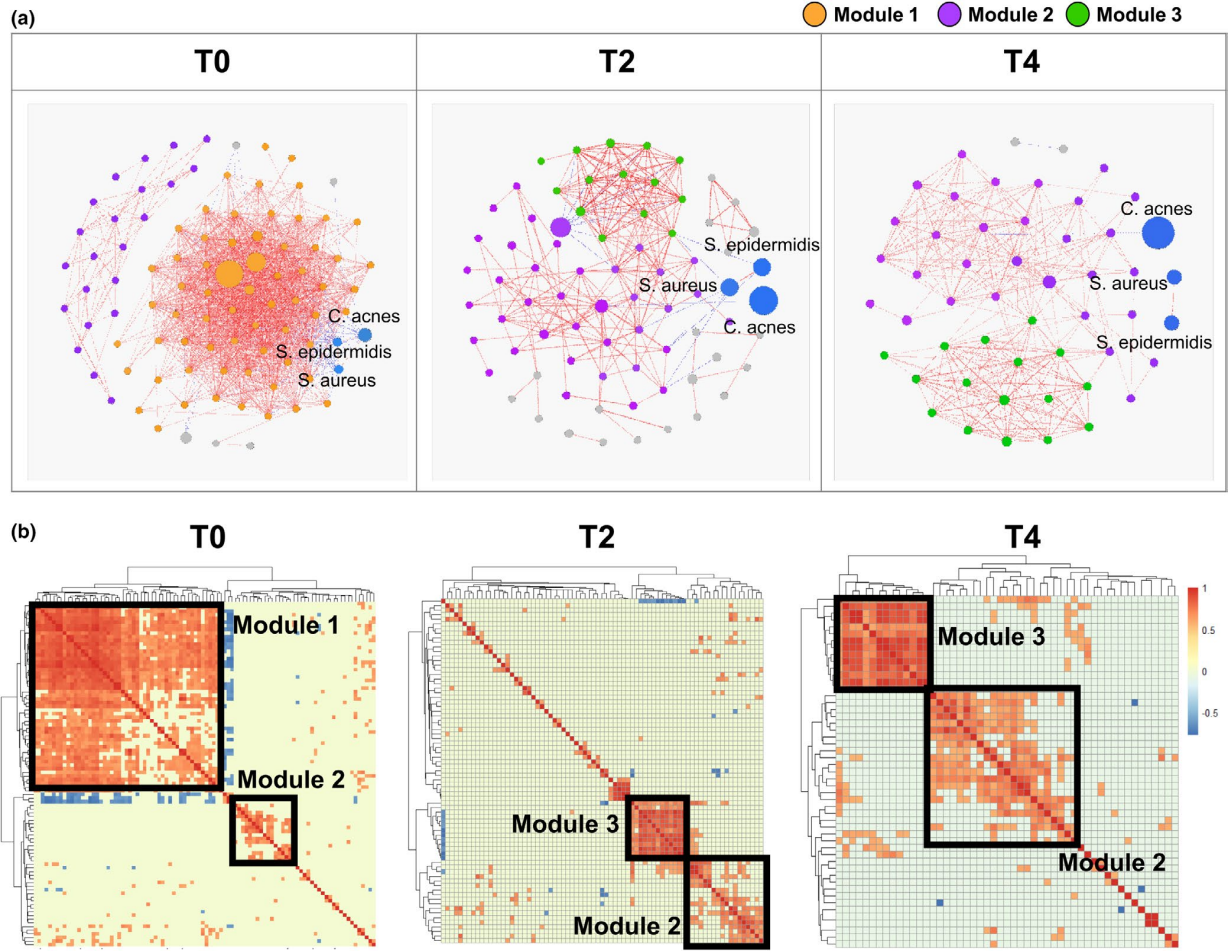
Changes to the bacterial network after use of the skincare product. (a) Bacterial co‐occurrence network. The size of each node is proportional to the relative abundance, node color represents bacterial module identity, and edge color indicates Spearman's correlation coefficient. (b) Correlation heatmap showing co‐occurrence patterns between bacteria

**FIGURE A2:**
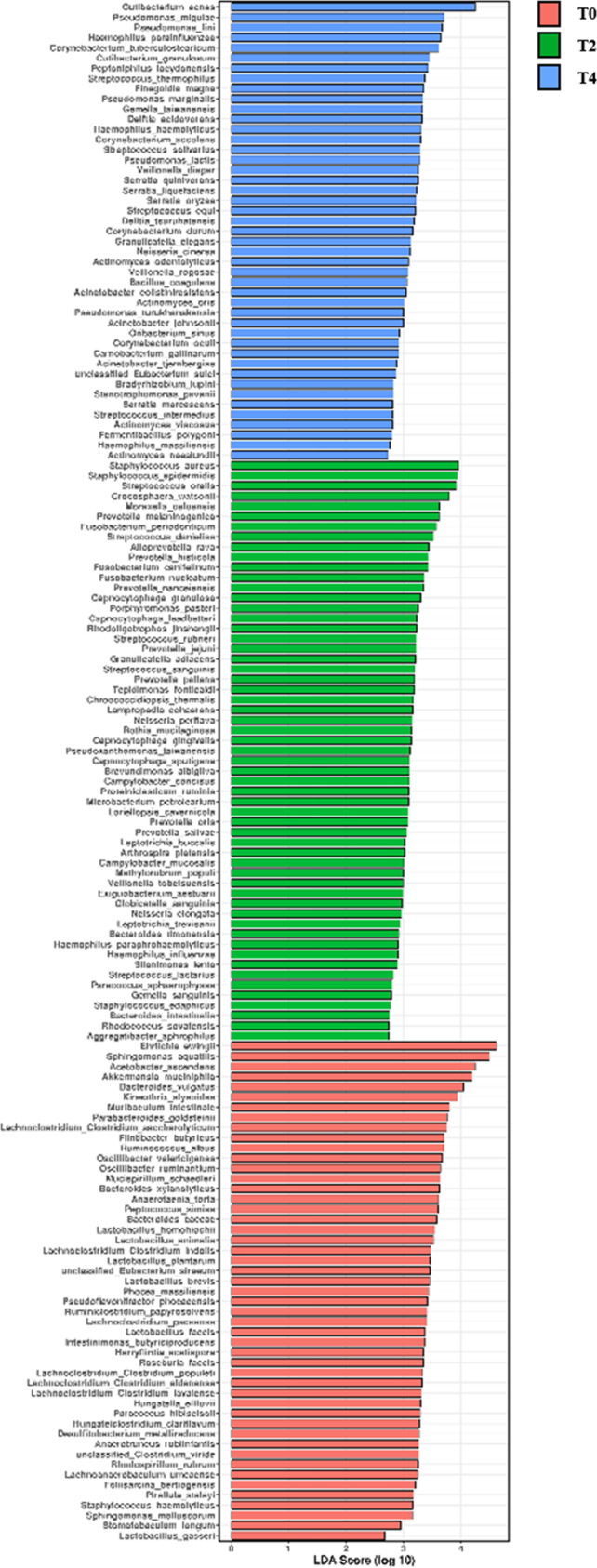
Linear discriminant analysis effect size (LEfSe) analysis of bacteria among sampling time points.

The correlation between microorganisms was investigated using bacterial co‐occurrence network analysis (Figure 2a). In the T0 bacterial co‐occurrence network, three OTUs labeled as *Cutibacterium acnes*, *Staphylococcus epidermidis*, and *Staphylococcus aureus* correlated negatively with the OTUs in the *Lachnoclostridium*, *Ehrlichia*, *Oscillibacter*, *Akkermansia*, and *Ruminococcus* genera. These genera were classified into the Clostridiales and Verrucomicrobiales orders and were largely found in skin samples before use of the skincare product (Figure A2). After its application, *Cutibacterium* and *Staphylococcus* OTUs became predominant in skin samples, and their negative edges in the T2 and T4 networks became smaller (Figure 2a).

The modularity of the bacterial co‐occurrence network was analyzed to investigate dynamic changes in overall skin microbial structures (Figure 2b). Three distinct clusters were identified in the network and their proportions varied following the use of the skincare product. Module 1 was composed of a number of OTUs including the orders Clostridiales and Verrucomicrobiales, but these OTUs disappeared in the T2 and T4 networks. Conversely, the proportion of module 2 (*Streptococcus*, *Neisseria*, and *Corynebacterium*) gradually increased from 23.4% in T0 to 50.67% in T2 and 58.82% in T4 samples. Module 3, which contained numerous *Pseudomonas* OTUs and a few *Actinomyces* OTUs, was found primarily in T2 (18.67%) and T4 (31.37%) samples. The OTUs in module 3 made up a small fraction of the T0 bacterial community and had little correlation to others (Figure 2b).

### 3.3 Effect of the skincare product on skin biophysical parameters

**FIGURE 3:**
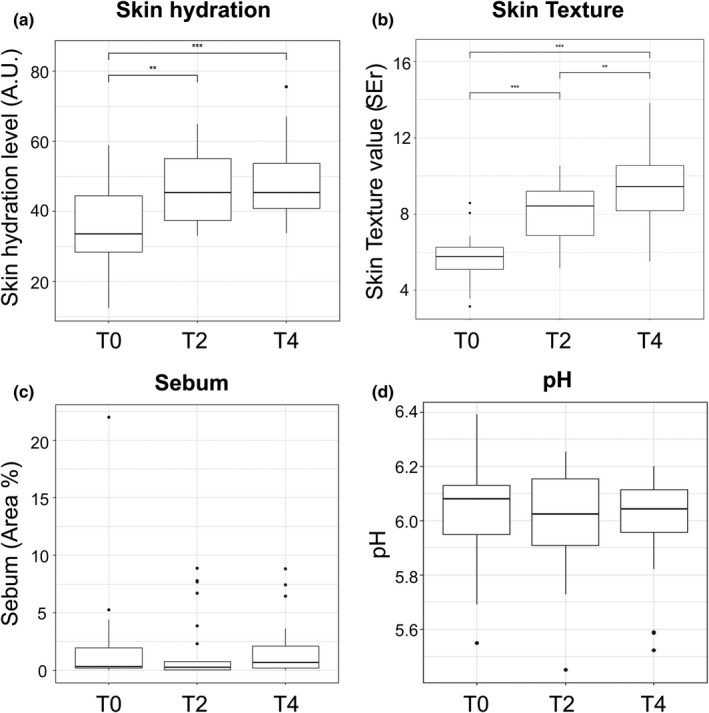
Comparison of skin biophysical parameters measured on the cheek. (a) Hydration level. (b) Texture level. (c) Sebum amount. (d) pH. **p < 0.01, ***p < 0.005

Every two weeks, biophysical parameters, including facial skin hydration, texture, sebum amount, and pH were measured (Figure 3). The skin hydration level was 36.15 ± 13.40 A.U. in T0 samples but increased significantly in T2 and T4 samples (46.55 ± 10.01 A.U. and 48.89 ± 11.29 A.U., respectively, Figure 3a). Skin texture gradually improved within the test period, going from 5.72 ± 1.22 SEr (T0) to 8.07 ± 1.44 SEr (T2) and 9.49 ± 1.93 SEr (T4) (Figure 3b). In comparison, sebum amount and pH remained almost identical throughout the evaluation period (Figure 3c,d).

### 3.4 Correlation between skin microbial community and skin biophysical parameters

**FIGURE 4:**
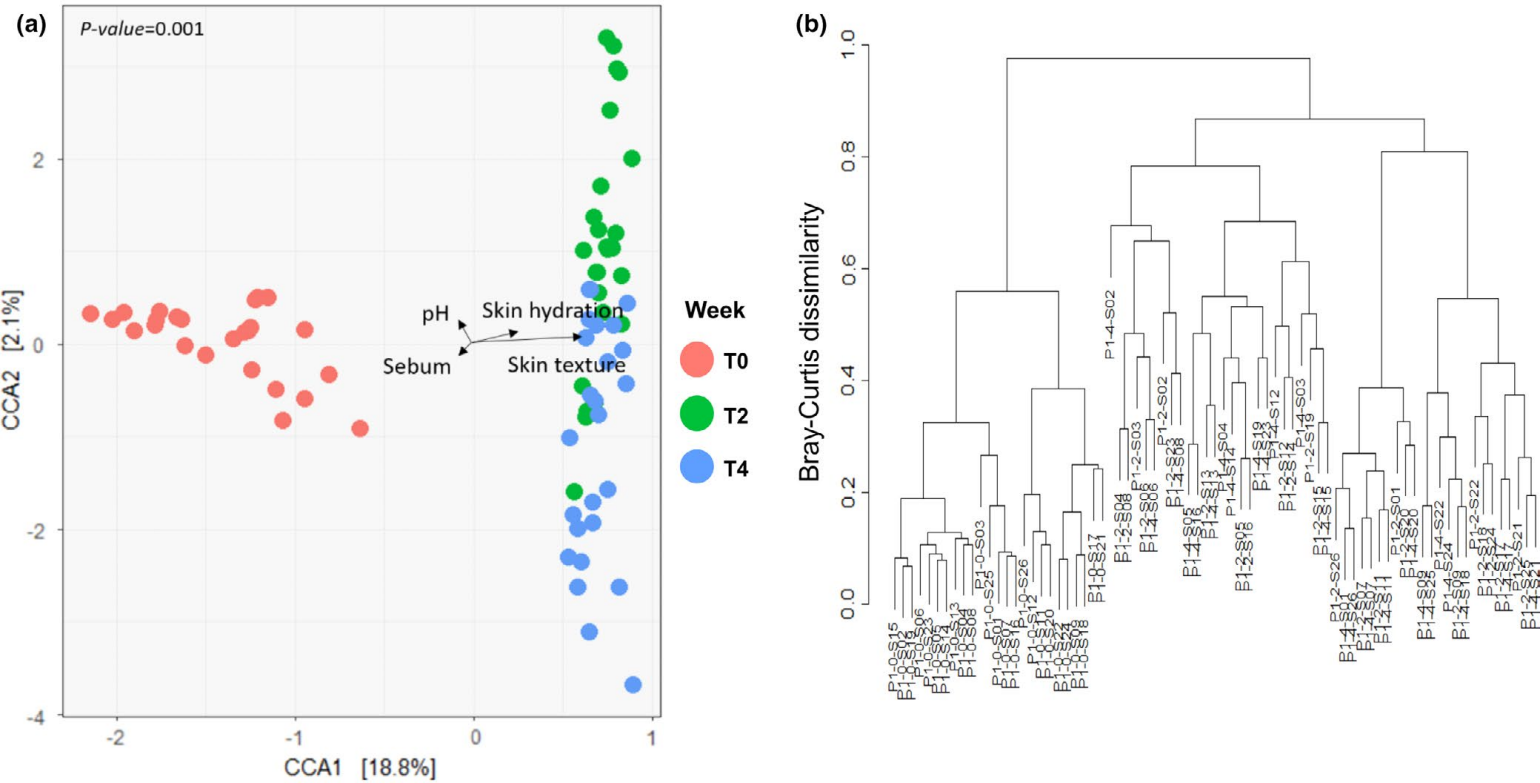
Dissimilarity of skin microbiota. (a) Canonical correspondence analysis (CCA) plot of skin microbiota. The points and arrows indicate each sample and skin biophysical parameters, respectively. (b) Clustering dendrogram of microbiota based on the Bray‐Curtis distance

CCA was used to investigate the correlation between the skin microbiome and skin biophysical parameters (Figure 4a). The total inertia of the CCA plot was 3.17; whereas the constrained inertia was 0.81, of which 18.8% was explained by the CCA1 axis and 2.1% by CCA2. In the CCA plot, the skin microbiomes after use of the skincare product (T2 and T4) were significantly separated from the initial skin microbiome (T0). A difference was evident also with respect to individual bacterial compositions (Figure 1c,d). T0 samples were clearly separated from other samples also in the cluster dendrogram (Figure 4b). Arrows on the CCA plot indicate the influence of different biophysical parameters on plot dispersion. Accordingly, skin hydration and texture had a more significant effect on the dispersion of the skin microbiome than sebum and pH.

**FIGURE 5:**
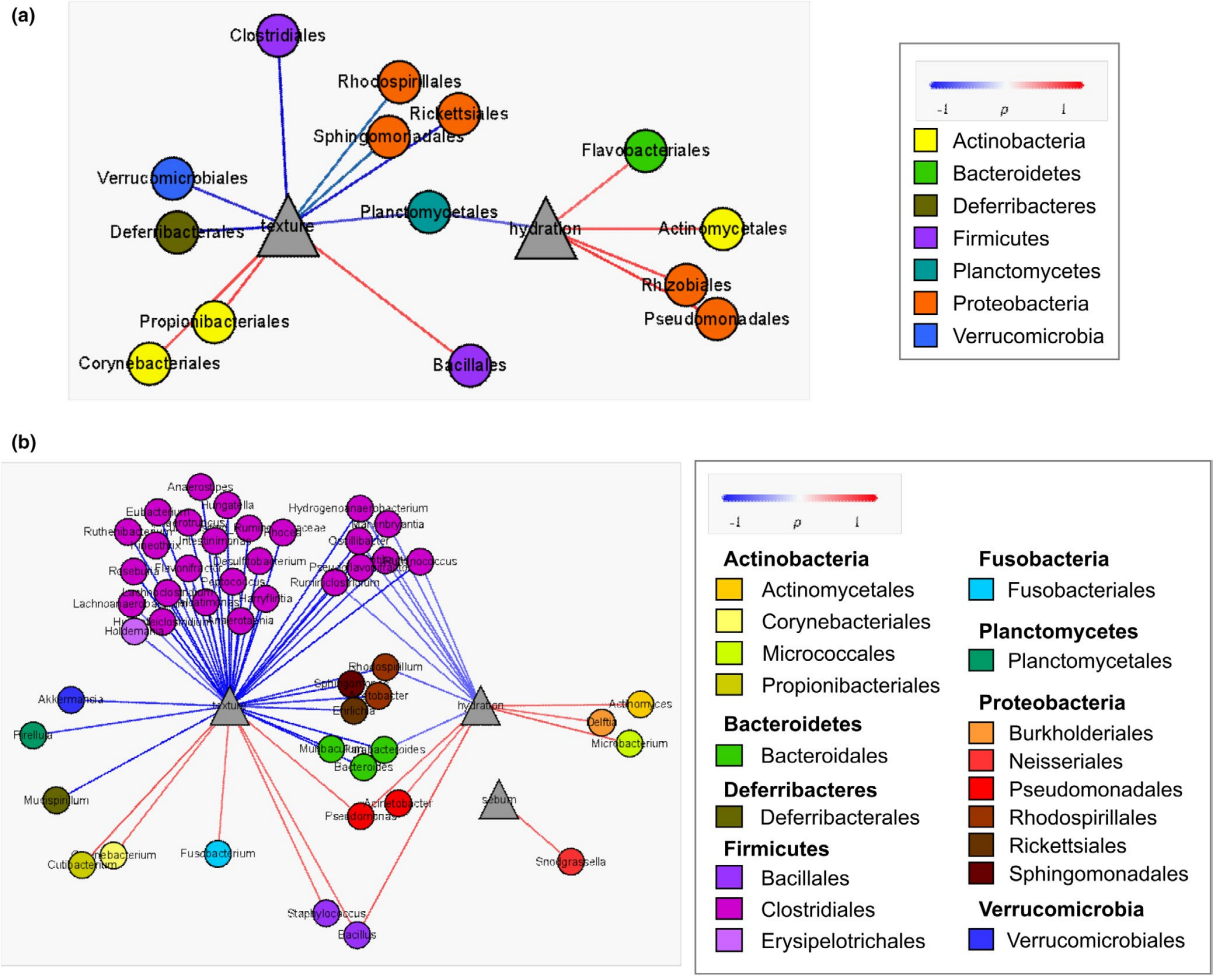
Correlation between skin microbiota and skin biophysical parameters. (a) Order level. (b) Genus level. Node colors in each network correspond to (a) phylum and (b) order level. Edge colors indicate Spearman's correlation coefficient

To investigate the exact relationship between skin microbial composition and its biophysical parameters, Spearman's correlation matrix was applied (Figure 5). In the correlation network, the occurrence of Propionibacteriales, Corynebacteriales, and Bacillaceae orders was associated with improved skin texture (*r* = 0.49, 0.47, and 0.76, respectively, *p* < 0.05, Figure 5a). In contrast, Clostridiales (*r* = −0.63) and Verrucomicrobiales (*r* = −0.53), both of which were members of module 1 in Figure 2b, correlated negatively with skin texture. Pseudomonadales and Actinomyces were related to skin hydration (*r* = 0.52, 0.43, respectively, *p* < 0.05). As shown in Figure 5b, the correlations between skin biophysical parameters and genera were mostly consistent with those at the order level. While *Snodgrassella*, which was not detected at the order level, was associated with sebum content (*r* = 0.46).

## 4 DISCUSSION

The present study indicates that daily use of the skincare product could affect the skin microbiome. Shannon diversity increased after the use of the skincare product (Figure 1b), confirming an earlier finding by Ciardiello et al. (2020), who revealed that the use of cosmetics elevated skin microbial alpha diversity (Kong et al., 2012). High alpha diversity is considered a hallmark of a healthy skin microbiome, as indicated by lower alpha diversity in damaged (Grice & Segre, 2011) and aged (Kim et al., 2019) skin. Therefore, according to our results, the skincare product might improve the microbial health of facial skin.

Significant alterations in the major genera composing the skin microbiome were found after skincare product application (Figure 1d, Figure A1). Whereas *Ehrlichia* and *Sphingomonas* were the most abundant genera in T0 samples, *Propionibacterium*, *Staphylococcus*, *Snodgrassella*, and *Streptococcus* were dominant in T2 and T4 samples. Indeed, these four genera are very common on human skin (Grice et al., 2009; Kim et al., 2019; Park et al., 2020). As a result, the skincare product appears to regulate the skin microbiome composition.

*Cutibacterium* and *Staphylococcus* displayed significant negative correlations with genera from the order Clostridiales and Verrucomicrobiales in the T0 bacterial network. Whereas the former became more abundant after the use of the skincare product, the proportion of Clostridiales and Verrucomicrobiales decreased (Figure 2a, Figure A1). Naik et al. showed the importance of microbial interactions among skin commensals for the development of skin immunity (Naik et al., 2012). In particular, *C*. *acnes* and *S*. *epidermidis* are known to prevent pathogenic bacterial colonization (Cogen et al., 2008; Fournière et al., 2020). More specifically, these two strains can directly inhibit pathogen growth by producing bacteriocins and competing for nutrients with other bacteria (Sanford & Gallo, 2013). Moreover, *C*. *acnes* and *S*. *epidermidis* can stimulate human keratinocytes and sebocytes to produce antimicrobial peptides and maintain a balanced skin microbiome (Gallo & Nakatsuji, 2011). Consequently, we hypothesize that the skincare product upheld the microbial equilibrium by favoring the growth of *Cutibacterium* and *Staphylococcus*. Notably, the negative correlation in network analysis did not represent direct competition among microbiota, and the relative abundance of these taxa varied among participants. To overcome this inherent limitation of network analysis, future studies should investigate direct interactions among microorganisms via co‐culture experiments.

Generally, modularity in a co‐occurrence network refers to phylogenetically close species or microbes inhabiting similar habitats (Olesen et al., 2007), and may disclose functional roles in bacterial ecosystems (Lurgi et al., 2019). Modules appearing in a specific environment are thought to exert specific functions, explaining why a shift in gut bacterial clusters might affect human health (Baldassano & Bassett, 2016; Liu et al., 2019). In the present study, the abundance of modules 2 and 3 gradually increased following the application of the skincare product (Figure 2b). Therefore, we conclude that the skincare product created an environment optimized for the growth of these modules. Moreover, microorganisms belonging to modules 2 and 3 might exert specific functions in balancing the skin ecosystem. The shift in bacterial modules caused by the use of skincare products has not been investigated extensively. Further studies should be performed to clarify the function of microbes in such modules and, specifically, how they affect the human skin.

The skin environment, defined by its hydration level, smoothness, sebum secretion, and pH, tends to affect skin health (Mukherjee et al., 2016). Dry skin is known to cause skin irritation and aging (Flynn et al., 2001; Rawlings & Matts, 2005), and elevated pH can lead to skin disease (Youn et al., 2013). Among various skin biophysical parameters, hydration level and smoothness were gradually improved after the use of the skincare product, while pH and sebum content were maintained (Figure 3). These results suggest that using skincare products might preserve skin health.

The correlation between skin microbial community and skin biophysical parameters revealed that hydration and texture were related to skin bacterial community composition (Figure 4a), with some orders and genera displaying significant correlations (Figure 5). Chang et al. suggested that the order level was the most appropriate for selecting a microbial indicator representative of a specific environment (Chang et al., 2017). The Actinomycetales and Pseudomonadales orders belonging to module 3 correlated significantly with skin hydration levels (Figure 2b, Figure 5a). Accordingly, we speculate that microorganisms belonging to module 3 grew well on hydrated skin, even though the function and role of these bacteria on the face have not been characterized yet. The genus *Delftia* also exhibited a significant correlation with skin hydration (*r* = 0.43, Figure 5b), which is consistent with previous findings (Wallen‐Russell, 2019). *Delftia* and *Ralstonia*, two genera of the Burkholderiales, have been reported as major bacterial taxa on the human face and forearm (Gao et al., 2007; Grice et al., 2008). Nevertheless, further studies are required to understand the relationship between these bacteria and skin hydration.

Various studies have investigated whether cosmetics could affect skin commensal microbes (Bouslimani et al., 2019; Wallen‐Russell, 2019; Williams & Gallo, 2015; Xu et al., 2020). Lee et al. showed that certain bacteria grew by metabolizing cosmetic ingredients (Lee et al., 2018). Here, we found that the use of a skincare product could improve the skin environment, as well as change its microbiome structure. In particular, the appearance and rise of specific bacterial modules, and their relationship with skin biophysical parameters were observed. These microorganisms can be used as bio‐indicators of facial skin conditions and a healthy skin microbial ecosystem. Furthermore, additional studies should be conducted to identify the main components or materials that are associated with changes in the skin microbiome and biophysical parameters.

## CONFLICT OF INTEREST

All authors are employees of LG Household and Health Care.

## AUTHOR CONTRIBUTIONS

**Bo Kyoung Hwang:** Conceptualization (equal); Data curation (lead); Methodology (supporting); Writing‐original draft (equal); Writing‐review & editing (equal). **Sado Lee:** Conceptualization (supporting); Data curation (supporting); Investigation (equal); Methodology (equal). **Joonoh Myoung:** Data curation (supporting); Investigation (equal). **Seung Jin Hwang:** Conceptualization (supporting); Methodology (equal); Project administration (equal); Supervision (supporting); Writing‐review & editing (supporting). **Jun Man Lim:** Project administration (supporting); Supervision (equal). **Eui Taek Jeong:** Methodology (equal). **Sun Gyoo Park:** Supervision (equal). **Sung Hun Youn:** Conceptualization (equal); Data curation (supporting); Methodology (equal); Project administration (equal); Supervision (equal); Writing‐original draft (equal); Writing‐review & editing (equal).

## ETHICS STATEMENT

The study was conducted in compliance with the Declaration of Helsinki and was approved by the Institutional Review Board of the SKINMED Clinical Trials Center (IRB certification number: SM‐CTCLG‐NOV19‐1).

## Data Availability

The raw sequence data are available in the NCBI repository under accession number PRJEB44885: https://www.ncbi.nlm.nih.gov/bioproject/PRJEB44885

## 1

**TABLE A1:** List of ingredients of su:m37° Secret Essence (LG Household & Health Care Ltd)

Classification	Ingredients (INCI)
Chemical	1,2‐Hexanediol, Acrylates/C10‐30 Alkyl Acrylate Crosspolymer, Allantoin, Anthemis Nobilis Flower Oil, Butylene Glycol, Ceramide NP, Cetearyl Alcohol, Cholesterol, Citral, Citronellol, Citrus Aurantifolia (Lime) Oil, Citrus Aurantium Bergamia (Bergamot) Fruit Oil, Dipropylene Glycol, Ferulic Acid, Geraniol, Glycereth−26, Glycerin, Hydrogenated Lecithin, Hydroxyethylcellulose, Lauryl Dimethicone/Polyglycerin−3 Crosspolymer, Lavandula Angustifolia (Lavender) Oil, Limonene, Linalool, Linoleic Acid, Methyl Gluceth−20, Niacinamide, Palmitic Acid, Panthenol, PEG/PPG−17/6 Copolymer, Pelargonium Graveolens Flower Oil, Poloxamer 234, Polymethylsilsesquioxane, Polyquaternium−51, Propanediol, Serine, Sucrose Stearate, Tromethamine, Water
Ferment	Lactobacillus Ferment Lysate, Lactobacillus/Panax Ginseng Root Ferment Filtrate, Lactobacillus/Pear Juice Ferment Filtrate, Lactobacillus/Rye Flour Ferment, Lactobacillus/Soybean Ferment Extract, Lactobacillus/Wasabia Japonica Root Ferment Extract, Lactobacillus/Water Hyacinth Ferment, Monascus/Rice Ferment, Saccharomyces/Barley Seed Ferment Filtrate, Saccharomyces/Grape Ferment Extract, Saccharomyces/Potato Extract Ferment Filtrate, Saccharomyces/Xylinum/Black Tea Ferment, Vinegar
Extract	Acer Palmatum Leaf Extract, Acorus Calamus Root Extract, Actinidia Polygama Fruit Extract, Akebia Quinata Extract, Allium Cepa (Onion) Bulb Extract, Allium Sativum (Garlic) Bulb Extract, Anemarrhena Asphodeloides Root Extract, Artemisia Princeps Leaf Extract, Avena Sativa (Oat) Kernel Extract, Baptisia Tinctoria Root Extract, Beta Vulgaris (Beet) Root Extract, Brassica Oleracea Capitata (Cabbage) Leaf Extract, Brassica Rapa (Turnip) Leaf Extract, Calendula Officinalis Flower Extract, Camellia Japonica Leaf Extract, Carica Papaya (Papaya) Fruit Extract, Carthamus Tinctorius (Safflower) Flower Extract, Cassia Obtusifolia Seed Extract, Centella Asiatica Extract, Chaenomeles Sinensis Fruit Extract, Chamomilla Recutita (Matricaria) Flower Extract, Cichorium Intybus (Chicory) Root Extract, Citrullus Lanatus (Watermelon) Fruit Extract, Citrus Aurantium Bergamia (Bergamot) Leaf Extract, Citrus Junos Fruit Extract, Citrus Unshiu Peel Extract, Codonopsis Lanceolata Root Extract, Crataegus Cuneata Fruit Extract, Cucumis Sativus (Cucumber) Fruit Extract, Curcuma Longa (Turmeric) Root Extract, Daucus Carota Sativa (Carrot) Root Extract, Diospyros Kaki Fruit Extract, Equisetum Arvense Extract, Eriobotrya Japonica Leaf Extract, Ficus Carica (Fig) Fruit Extract, Geranium Robertianum Extract, Ginkgo Biloba Nut Extract, Glechoma Hederacea Extract, Glycyrrhiza Glabra (Licorice) Root Extract, Grifola Frondosa Fruiting Body Extract, Helianthus Annuus (Sunflower) Seed Extract, Hemerocallis Fulva Flower Extract, Houttuynia Cordata Extract, Ipomoea Batatas Root Extract, Lentinus Edodes Extract, Lippia Citriodora Leaf Extract, Lonicera Japonica (Honeysuckle) Flower Extract, Lycium Chinense Fruit Extract, Melissa Officinalis Extract, Mentha Piperita (Peppermint) Flower/Leaf/Stem Extract, Morus Bombycis Leaf Extract, Musa Sapientum (Banana) Fruit Extract, Nelumbo Nucifera Root Extract, Nepeta Cataria Extract, Ocimum Basilicum (Basil) Leaf Extract, Panicum Miliaceum (Millet) Seed Extract, Perilla Frutescens Leaf Extract, Phaseolus Angularis Seed Extract, Plantago Major Seed Extract, Platycodon Grandiflorus Root Extract, Portulaca Oleracea Extract, Prunus Armeniaca (Apricot) Fruit Extract, Prunus Persica (Peach) Fruit Extract, Prunus Salicina Fruit Extract, Pyrus Malus (Apple) Fruit Extract, Raphanus Sativus (Radish) Root Extract, Rehmannia Glutinosa Root Extract, Rosmarinus Officinalis (Rosemary) Leaf Extract, Rubus Idaeus (Raspberry) Fruit Extract, Rubus Idaeus (Raspberry) Leaf Extract, Salvia Officinalis (Sage) Leaf Extract, Sasa Veitchii Leaf Extract, Schizonepeta Tenuifolia Extract, Sesamum Indicum (Sesame) Seed Extract, Spinacia Oleracea (Spinach) Leaf Extract, Spiraea Ulmaria Extract, Stellaria Media (Chickweed) Extract, Taraxacum Officinale (Dandelion) Leaf Extract, Thymus Vulgaris (Thyme) Leaf Extract, Trichosanthes Kirilowii Root Extract, Undaria Pinnatifida Extract, Vanilla Tahitensis Fruit Extract, Wine Extract, Zingiber Officinale (Ginger) Root Extract

**TABLE A2:** Pyrosequencing data and statistical analysis of 16S rRNA genes of 75 skin samples

Sample ID	Count reads			Observed OTUs			Shannon diversity		
	0_week	2_week	4_week	0_week	2_week	4_week	0_week	2_week	4_week
P1_S01	135,224	86,249	164,105	835	777	639	5.662742	6.455305	5.203586
P1_S02	132,078	78,042	139,884	897	814	745	5.457821	7.482586	5.813859
P1_S03	103,746	78,457	120,699	1,021	868	749	6.562266	7.404397	7.009846
P1_S04	107,214	70,919	124,288	932	918	849	5.876826	7.214149	7.099387
P1_S05	163,619	74,966	147,215	841	735	686	5.516962	6.448567	5.582202
P1_S06	128,909	70,163	146,208	951	815	839	5.777814	6.218802	7.099817
P1_S07	125,459	97,927	149,843	896	795	714	5.631805	6.203497	5.576054
P1_S08	122,896	102,869	121,394	907	857	850	5.869186	7.189305	7.209324
P1_S09	80,455	70,782	207,230	803	674	767	5.074643	4.731227	4.729183
P1_S11	91,984	75,318	213,198	787	628	775	5.799308	6.071564	6.051984
P1_S12	87,210	78,060	107,374	817	705	652	5.745201	7.460329	6.96595
P1_S13	83,513	88,177	166,869	834	852	806	5.633245	6.970269	6.329246
P1_S14	87,798	68,769	194,289	829	692	791	5.581537	6.954256	6.99845
P1_S15	113,063	106,672	178,452	669	766	837	5.008865	7.804252	7.802099
P1_S16	84,030	96,512	196,342	902	676	747	5.711661	6.558116	5.666585
P1_S17	98,626	66,246	151,521	1,001	781	907	6.263955	6.066502	6.402953
P1_S18	97,086	98,475	190,749	890	787	725	5.565942	6.105868	4.370777
P1_S19	105,229	100,472	157,842	701	685	723	5.309526	7.320767	6.795368
P1_S20	103,923	89,203	157,188	910	823	857	6.049413	6.890482	6.643334
P1_S21	79,104	76,648	158,544	692	548	611	5.006225	4.652727	4.894047
P1_S22	114,420	86,386	209,050	701	773	885	5.611431	6.300585	5.193767
P1_S23	106,245	91,472	169,539	807	788	738	5.553106	7.34191	6.859204
P1_S24	98,055	84,040	151,009	809	793	768	5.443179	5.746173	4.602597
P1_S25	97,385	96,221	161,791	742	672	740	5.612869	5.738126	4.83177
P1_S26	142,039	80,304	185,322	846	753	741	5.780286	5.96931	5.242071

## References

[mbo31236-bib-0001] Baldassano, S. N. , & Bassett, D. S. (2016). Topological distortion and reorganized modular structure of gut microbial co‐occurrence networks in inflammatory bowel disease. Scientific Reports, 6, 26087. 10.1038/srep26087. 27188829 PMC4870640

[mbo31236-bib-0002] Bastian, M. , Heymann, S. , & Jacomy, M. (2009). Gephi: An open source software for exploring and manipulating networks. In International AAAI conference on weblogs and social media.

[mbo31236-bib-0003] Bouslimani, A. , da Silva, R. , Kosciolek, R. , Janssen, S. , Callewaert, C. , Amir, A. , Dorrestein, K. , Melnik, A. V. , Zaramela, L. S. , Kim, J. N. , Humphrey, G. , Schwartz, T. , Sanders, K. , Brennan, C. , Luzzatto‐Knaan, T. , Ackermann, G. , McDonald, D. , Zengler, K. , Knight, R. , & Dorrestein, P. C. (2019). The impact of skin care products on skin chemistry and microbiome dynamics. BMC Biology, 17, 47. 10.1186/s12915-019-0660-6. 31189482 PMC6560912

[mbo31236-bib-0004] Byrd, A. L. , Belkaid, Y. , & Segre, J. A. (2018). The human skin microbiome. Nature Reviews Microbiology, 16, 143–155. 10.1038/nrmicro.2017.157. 29332945

[mbo31236-bib-0005] Caporaso, J. G. , Bittinger, K. , Bushman, F. D. , DeSantis, T. Z. , Andersen, G. L. , & Knight, R. (2010). PyNAST: A flexible tool for aligning sequences to a template alignment. Bioinformatics, 26, 266–267. 10.1093/bioinformatics/btp636. 19914921 PMC2804299

[mbo31236-bib-0006] Caporaso, J. G. , Lauber, C. L. , Walters, W. A. , Berg‐Lyons, D. , Huntley, J. , Fierer, N. , Owens, S. M. , Betley, J. , Fraser, L. , Bauer, M. , Gormley, N. , Gilbert, J. A. , Smith, G. , & Knight, R. (2012). Ultra‐high‐throughput microbial community analysis on the Illumina HiSeq and MiSeq platforms. ISME Journal, 6, 1621–1624. 10.1038/ismej.2012.8. PMC3400413 22402401

[mbo31236-bib-0007] Chang, H. X. , Haudenshield, J. S. , Bowen, C. R. , & Hartman, G. L. (2017). Metagenome‐wide association study and machine learning prediction of bulk soil microbiome and crop productivity. Frontiers in Microbiology, 8, 519. 10.3389/fmicb.2017.00519. 28421041 PMC5378059

[mbo31236-bib-0008] Chen, T. , Yu, W. H. , Izard, J. , Baranova, O. V. , Lakshmanan, A. , & Dewhirst, F. E. (2010). The Human Oral Microbiome Database: a web accessible resource for investigating oral microbe taxonomic and genomic information. Database, 2010(0), baq013. 20624719 10.1093/database/baq013 PMC2911848

[mbo31236-bib-0009] Ciardiello, T. , Pinto, D. , Marotta, L. , Giuliani, G. , & Rinaldi, F. (2020). Effects of fermented oils on alpha‐biodiversity and relative abundance of cheek resident skin microbiota. Cosmetics, 7, 34. 10.3390/cosmetics7020034.

[mbo31236-bib-0010] Cogen, A. L. , Nizet, V. , & Gallo, R. L. (2008). Skin microbiota: A source of disease or defence? British Journal of Dermatology, 158, 442–455. 10.1111/j.1365-2133.2008.08437.x. PMC2746716 18275522

[mbo31236-bib-0011] Dimitriu, P. A. , Iker, B. , Malik, K. , Leung, H. , Mohn, W. W. , & Hillebrand, G. G. (2019). New insights into the intrinsic and extrinsic factors that shape the human skin microbiome. mBio, 10(4), e00839–e00919. 10.1128/mBio.00839-19. 31266865 PMC6606800

[mbo31236-bib-0012] Edgar, R. C. (2010). Search and clustering orders of magnitude faster than BLAST. Bioinformatics, 26, 2460–2461. 10.1093/bioinformatics/btq461. 20709691

[mbo31236-bib-0013] Edgar, R. C. (2017). SEARCH_16S: A new algorithm for identifying 16S ribosomal RNA genes in contigs and chromosomes. bioRxiv, 124131.

[mbo31236-bib-0014] Findley, K. , Oh, J. , Yang, J. , Conlan, S. , Deming, C. , Meyer, J. A. , Schoenfeld, D. , Nomicos, E. , Park, M. , Intramural Sequencing Center Comparative Sequencing Program, N. I. H. , Kong, H. H. , & Segre, J. A. (2013). Topographic diversity of fungal and bacterial communities in human skin. Nature, 498, 367–370. 10.1038/nature12171. 23698366 PMC3711185

[mbo31236-bib-0015] Flores, G. E. , Caporaso, J. G. , Henley, J. B. , Rideout, J. R. , Domogala, D. , Chase, J. , Leff, J. W. , Vázquez‐Baeza, Y. , Gonzalez, A. , Knight, R. , Dunn, R. R. , & Fierer, N. (2014). Temporal variability is a personalized feature of the human microbiome. Genome Biology, 15, 531. 10.1186/s13059-014-0531-y. 25517225 PMC4252997

[mbo31236-bib-0016] Flynn, T. C. , Petros, J. , Clark, R. E. , & Viehman, G. E. (2001). Dry skin and moisturizers. Clinics in Dermatology, 19, 387–392. 10.1016/S0738-081X(01)00199-7. 11535378

[mbo31236-bib-0017] Fournière, M. , Latire, T. , Souak, D. , Feuilloley, M. G. J. , & Bedoux, G. (2020). *Staphylococcus epidermidis* and *Cutibacterium acnes*: Two major sentinels of skin microbiota and the influence of cosmetics. Microorganisms, 8, 1752. 10.3390/microorganisms8111752. PMC7695133 33171837

[mbo31236-bib-0018] Gallo, R. L. , & Nakatsuji, T. (2011). Microbial symbiosis with the innate immune defense system of the skin. Journal of Investigative Dermatology, 131, 1974–1980. 10.1038/jid.2011.182. PMC3174284 21697881

[mbo31236-bib-0019] Gao, Z. , Tseng, C. H. , Pei, Z. , & Blaser, M. J. (2007). Molecular analysis of human forearm superficial skin bacterial biota. Proceedings of the National Academy of Sciences, USA, 104, 2927–2932. 10.1073/pnas.0607077104. PMC1815283 17293459

[mbo31236-bib-0020] Gotelli, N. J. , & Colwell, R. K. (2001). Quantifying biodiversity: Procedures and pitfalls in the measurement and comparison of species richness. Ecology Letters, 4, 379–391. 10.1046/j.1461-0248.2001.00230.x.

[mbo31236-bib-0021] Grice, E. A. , Kong, H. H. , Conlan, S. , Deming, C. B. , Davis, J. , Young, A. C. , Comparative Sequencing Program, N. I. S. C. , Bouffard, G. G. , Blakesley, R. W. , Murray, P. R. , Green, E. D. , Turner, M. L. , & Segre, J. A. (2009). Topographical and temporal diversity of the human skin microbiome. Science, 324, 1190–1192. 10.1126/science.1171700. 19478181 PMC2805064

[mbo31236-bib-0022] Grice, E. A. , Kong, H. H. , Renaud, G. , Young, A. C. , Comparative Sequencing Program, N. I. S. C. , Bouffard, G. G. , Blakesley, R. W. , Wolfsberg, T. G. , Turner, M. L. , & Segre, J. A. (2008). A diversity profile of the human skin microbiota. Genome Research, 18, 1043–1050. 10.1101/gr.075549.107. 18502944 PMC2493393

[mbo31236-bib-0023] Grice, E. A. , & Segre, J. A. (2011). The skin microbiome. Nature Reviews Microbiology, 9, 244–253. 10.1038/nrmicro2537. 21407241 PMC3535073

[mbo31236-bib-0024] Hillebrand, G. G. , Dimitriu, P. , Malik, K. M. , Park, Y. , Qu, D. , Mohn, W. W. , & Kong, R. (2021). Temporal variation of the facial skin microbiome: A 2‐year longitudinal study in healthy adults. Plastic and Reconstructive Surgery, 147, 50S–61S. 10.1097/PRS.0000000000007621. 33347075

[mbo31236-bib-0025] Kim, H. J. , Kim, H. , Kim, J. J. , Myeong, N. R. , Kim, T. , Park, T. , Kim, E. , Choi, J. Y. , Lee, J. , An, S. , & Sul, W. J. (2018). Fragile skin microbiomes in megacities are assembled by a predominantly niche‐based process. Science Advances, 4, e1701581. 10.1126/sciadv.1701581. 29532031 PMC5842045

[mbo31236-bib-0026] Kim, H. J. , Kim, J. J. , Myeong, N. R. , Kim, T. , Kim, D. , An, S. , Kim, H. , Park, T. , Jang, S. I. , Yeon, J. H. , Kwack, I. , & Sul, W. J. (2019). Segregation of age‐related skin microbiome characteristics by functionality. Scientific Reports, 9, 1–11. 10.1038/s41598-019-53266-3. 31727980 PMC6856112

[mbo31236-bib-0027] Kong, H. H. (2011). Skin microbiome: Genomics‐based insights into the diversity and role of skin microbes. Trends in Molecular Medicine, 17, 320–328. 10.1016/j.molmed.2011.01.013. 21376666 PMC3115422

[mbo31236-bib-0028] Kong, H. H. , Oh, J. , Deming, C. , Conlan, S. , Grice, E. A. , Beatson, M. A. , Nomicos, E. , Polley, E. C. , Komarow, H. D. , Comparative Sequence Program, N. I. S. C. , Murray, P. R. , Turner, M. L. , & Segre, J. A. (2012). Temporal shifts in the skin microbiome associated with disease flares and treatment in children with atopic dermatitis. Genome Research, 22, 850–859. 10.1101/gr.131029.111. 22310478 PMC3337431

[mbo31236-bib-0029] Kuczynski, J. , Stombaugh, J. , Walters, W. A. , González, A. , Caporaso, J. G. , & Knight, R. (2012). Using QIIME to analyze 16S rRNA gene sequences from microbial communities. Current Protocols in Microbiology, 1, Unit‐1E.5. 10.1002/9780471729259.mc01e05s27. PMC4477843 23184592

[mbo31236-bib-0030] Lee, H. J. , Jeong, S. E. , Lee, S. , Kim, S. , Han, H. , & Jeon, C. O. (2018). Effects of cosmetics on the skin microbiome of facial cheeks with different hydration levels. MicrobiologyOpen, 7, e00557. 10.1002/mbo3.557. 29193830 PMC5911989

[mbo31236-bib-0031] Leung, M. H. Y. , Tong, X. , Bastien, P. , Guinot, F. , Tenenhaus, A. , Appenzeller, B. M. R. , Betts, R. J. , Mezzache, S. , Li, J. , Bourokba, N. , Breton, L. , Clavaud, C. , & Lee, P. K. H. (2020). Changes of the human skin microbiota upon chronic exposure to polycyclic aromatic hydrocarbon pollutants. Microbiome, 8, 100. 10.1186/s40168-020-00874-1. 32591010 PMC7320578

[mbo31236-bib-0032] Li, M. , Budding, A. E. , van der Lugt‐Degen, M. , Du‐Thumm, L. , Vandeven, M. , & Fan, A. (2019). The influence of age, gender and race/ethnicity on the composition of the human axillary microbiome. International Journal of Cosmetic Science, 41, 371–377. 10.1111/ics.12549. 31190339

[mbo31236-bib-0033] Liu, F. , Li, Z. , Wang, X. , Xue, C. , Tang, Q. , & Li, R. W. (2019). Microbial co‐occurrence patterns and keystone species in the gut microbial community of mice in response to stress and chondroitin sulfate disaccharide. International Journal of Molecular Sciences, 20, 2130. 10.3390/ijms20092130. PMC6539173 31052157

[mbo31236-bib-0034] Lurgi, M. , Thomas, T. , Wemheuer, B. , Webster, N. S. , & Montoya, J. M. (2019). Modularity and predicted functions of the global sponge‐microbiome network. Nature Communications, 10, 992. 10.1038/s41467-019-08925-4. PMC6397258 30824706

[mbo31236-bib-0035] Mukherjee, S. , Mitra, R. , Maitra, A. , Gupta, S. , Kumaran, S. , Chakrabortty, A. , & Majumder, P. P. (2016). Sebum and hydration levels in specific regions of human face significantly predict the nature and diversity of facial skin microbiome. Scientific Reports, 6, 36062. 10.1038/srep36062. 27786295 PMC5081537

[mbo31236-bib-0036] Naik, S. , Bouladoux, N. , Wilhelm, C. , Molloy, M. J. , Salcedo, R. , Kastenmuller, W. , Deming, C. , Quinones, M. , Koo, L. , Conlan, S. , Spencer, S. , Hall, J. A. , Dzutsev, A. , Kong, H. , Campbell, D. J. , Trinchieri, G. , Segre, J. A. , & Belkaid, Y. (2012). Compartmentalized control of skin immunity by resident commensals. Science, 337, 1115–1119. 10.1126/science.1225152. 22837383 PMC3513834

[mbo31236-bib-0037] Navas‐Molina, J. A. , Peralta‐Sánchez, J. M. , González, A. , McMurdie, P. J. , Vázquez‐Baeza, Y. , Xu, Z. , Ursell, L. K. , Lauber, C. , Zhou, H. , Song, S. J. , Huntley, J. , Ackermann, G. L. , Berg‐Lyons, D. , Holmes, S. , Caporaso, J. G. , & Knight, R. (2013). Advancing our understanding of the human microbiome using QIIME. Methods in Enzymology, 531, 371–444. 24060131 10.1016/B978-0-12-407863-5.00019-8 PMC4517945

[mbo31236-bib-0038] Oh, J. , Byrd, A. L. , Park, M. , Comparative Sequencing Program, N. I. S. C. , Kong, H. H. , & Segre, J. A. (2016). Temporal stability of the human skin microbiome. Cell, 165, 854–866. 10.1016/j.cell.2016.04.008. 27153496 PMC4860256

[mbo31236-bib-0039] Olesen, J. M. , Bascompte, J. , Dupont, Y. L. , & Jordano, P. (2007). The modularity of pollination networks. Proceedings of the National Academy of Sciences, USA, 104, 19891–19896. 10.1073/pnas.0706375104. PMC2148393 18056808

[mbo31236-bib-0040] Park, S. Y. , Kim, H. S. , Lee, S. H. , & Kim, S. (2020). Characterization and analysis of the skin microbiota in acne: Impact of systemic antibiotics. Journal of Clinical Medicine, 9, 168. 10.3390/jcm9010168. PMC7019264 31936262

[mbo31236-bib-0041] Perez, G. I. P. , Gao, Z. , Jourdain, R. , Ramirez, J. , Gany, F. , Clavaud, C. , Demaude, J. , Breton, L. , & Blaser, M. J. (2016). Body site is a more determinant factor than human population diversity in the healthy skin microbiome. PLoS One, 11, e0151990. 10.1371/journal.pone.0151990. 27088867 PMC4835103

[mbo31236-bib-0042] Quast, C. , Pruesse, E. , Yilmaz, P. , Gerken, J. , Schweer, T. , Yarza, P. , Peplies, J. , & Glöckner, F. O. (2013). The SILVA ribosomal RNA gene database project: improved data processing and web‐based tools. Nucleic Acids Research, 41, D590–D596. 23193283 10.1093/nar/gks1219 PMC3531112

[mbo31236-bib-0043] Rawlings, A. V. , & Matts, P. J. (2005). Stratum corneum moisturization at the molecular level: An update in relation to the dry skin cycle. Journal of Investigative Dermatology, 124, 1099–1110. 10.1111/j.1523-1747.2005.23726.x. 15955083

[mbo31236-bib-0044] Rognes, T. , Flouri, T. , Nichols, B. , Quince, C. , & Mahé, F. (2016). VSEARCH: A versatile open source tool for metagenomics. PeerJ, 4, e2584. 10.7717/peerj.2584. 27781170 PMC5075697

[mbo31236-bib-0045] Ross, A. A. , Doxey, A. C. , & Neufeld, J. D. (2017). The skin microbiome of cohabiting couples. mSystems, 2(4), e00043–e00117. 10.1128/mSystems.00043-17. 28761935 PMC5527301

[mbo31236-bib-0046] Sanford, J. A. , & Gallo, R. L. (2013). Functions of the skin microbiota in health and disease. Seminars in Immunology, 25, 370–377. 10.1016/j.smim.2013.09.005. 24268438 PMC4219649

[mbo31236-bib-0047] Schommer, N. N. , & Gallo, R. L. (2013). Structure and function of the human skin microbiome. Trends in Microbiology, 21, 660–668. 10.1016/j.tim.2013.10.001. 24238601 PMC4744460

[mbo31236-bib-0048] Shannon, P. , Markiel, A. , Ozier, O. , Baliga, N. S. , Wang, J. T. , Ramage, D. , Amin, N. , Schwikowski, B. , & Ideker, T. (2003). Cytoscape: A software environment for integrated models of biomolecular interaction networks. Genome Research, 13, 2498–2504. 10.1101/gr.1239303. 14597658 PMC403769

[mbo31236-bib-0049] Shibagaki, N. , Suda, W. , Clavaud, C. , Bastien, P. , Takayasu, L. , Iioka, E. , Kurokawa, R. , Yamashita, N. , Hattori, Y. , Shindo, C. , Breton, L. , & Hattori, M. (2017). Aging‐related changes in the diversity of women’s skin microbiomes associated with oral bacteria. Scientific Reports, 7, 1–10. 10.1038/s41598-017-10834-9. 28874721 PMC5585242

[mbo31236-bib-0050] Soergel, D. A. , Dey, N. , Knight, R. , & Brenner, S. E. (2012). Selection of primers for optimal taxonomic classification of environmental 16S rRNA gene sequences. ISME Journal, 6, 1440–1444. 10.1038/ismej.2011.208. PMC3379642 22237546

[mbo31236-bib-0051] Two, A. M. , Nakatsuji, T. , Kotol, P. F. , Arvanitidou, E. , Du‐Thumm, L. , Hata, T. R. , & Gallo, R. L. (2016). The cutaneous microbiome and aspects of skin antimicrobial defense system resist acute treatment with topical skin cleansers. Journal of Investigative Dermatology, 136, 1950–1954. 10.1016/j.jid.2016.06.612. 27377698

[mbo31236-bib-0052] Wallen‐Russell, C. (2019). The role of every‐day cosmetics in altering the skin microbiome: A study using biodiversity. Cosmetics, 6, 2.

[mbo31236-bib-0053] Wang, Q. , Garrity, G. M. , Tiedje, J. M. , & Cole, J. R. (2007). Naïve Bayesian classifier for rapid assignment of rRNA sequences into the new bacterial taxonomy. Applied and Environmental Microbiology, 73, 5261–5267. 17586664 10.1128/AEM.00062-07 PMC1950982

[mbo31236-bib-0054] Williams, M. R. , & Gallo, R. L. (2015). The role of the skin microbiome in atopic dermatitis. Current Allergy and Asthma Reports, 15, 65. 10.1007/s11882-015-0567-4. 26404536

[mbo31236-bib-0055] Xu, Z. , Liu, X. , Niu, Y. , Shen, C. , Heminger, K. , Moulton, L. , Yu, A. , Allen, T. , Zhang, L. , Yue, F. , Liu, J. , Xu, Y. , Zhao, H. , Li, L. , Cambron, T. , Xu, J. , Smith, E. , & Wei, K. (2020). Skin benefits of moisturising body wash formulas for children with atopic dermatitis: A randomised controlled clinical study in China. Australasian Journal of Dermatology, 61, e54–e59. 10.1111/ajd.13153. 31512226

[mbo31236-bib-0056] Ying, S. , Zeng, D. N. , Chi, L. , Tan, Y. , Galzote, C. , Cardona, C. , Lax, S. , Gilbert, J. , & Quan, Z. X. (2015). The influence of age and gender on skin‐associated microbial communities in urban and rural human populations. PLoS One, 10, e0141842. 10.1371/journal.pone.0141842. 26510185 PMC4624872

[mbo31236-bib-0057] Youn, S. H. , Choi, C. W. , Choi, J. W. , & Youn, S. W. (2013). The skin surface pH and its different influence on the development of acne lesion according to gender and age. Skin Research and Technology, 19, 131–136. 10.1111/srt.12023. 23279122

